# Comparing health systems readiness for integrating domestic violence services in Brazil, occupied Palestinian Territories, Nepal and Sri Lanka

**DOI:** 10.1093/heapol/czae032

**Published:** 2024-05-04

**Authors:** Manuela Colombini, Satya Shrestha, Stephanie Pereira, Beatriz Kalichman, Prabhash Siriwardhana, Tharuka Silva, Rana Halaseh, Ana Flavia d’Oliveira, Poonam Rishal, Pusp Raj Bhatt, Amira Shaheen, Nagham Joudeh, Thilini Rajapakse, Abdulsalam Alkaiyat, Gene Feder, Claudia Garcia Moreno, Loraine J Bacchus

**Affiliations:** Department of Global Health and Development, London School of Hygiene and Tropical Medicine, London WC1H 9RA, United Kingdom; Kathmandu University School of Medical Sciences, Nepal and Faculty of Health Sciences, Dhulikhel 45209, Nepal; University of Bristol, Bristol BS81UD, United Kingdom; Faculty of Medicine, University of São Paulo Institute of Biomedical Sciences, Sao Paulo, Sao Paulo CEP 01246 903, Brazil; Department of Preventive Medicine, Faculty of Medicine, Sao Paulo CEP 01246 903, Brazil; Department of Social Sciences, Rajarata University of Sri Lanka, Mihintale 50300, Sri Lanka; South Asian Clinical Toxicology Research Collaboration, University of Peradeniya, Peradeniya 20400, Sri Lanka; Department of Psychiatry, Faculty of Medicine, Kandy, Sri Lanka; Occupied Palestinian Territories, An-Najah National University, Palestine; Faculty of Medicine, University of São Paulo Institute of Biomedical Sciences, Sao Paulo, Sao Paulo CEP 01246 903, Brazil; Kathmandu University School of Medical Sciences, Dhulikhel Hospital, Dhulikhel 45209, Nepal; Kathmandu University School of Medical Sciences, Dhulikhel Hospital, Dhulikhel 45209, Nepal; Faculty of Medicine and Health Sciences, Occupied Palestinian Territories, An-Najah National University, Nablus, Palestine; Faculty of Medicine and Health Sciences, An-Najah National University, Nablus, Palestine; Department of Psychiatry, Faculty of Medicine, Kandy, Sri Lanka; Faculty of Medicine and Health Sciences, Occupied Palestinian Territories, An-Najah National University, Nablus, Palestine; University of Bristol, Centre for Academic Primary Care, Bristol BS81UD, United Kingdom; Formerly at Department of Reproductive Health and Research, World Health Organisation, Geneva 1211, Switzerland; Department of Global Health and Development, London School of Hygiene and Tropical Medicine, London WC1H 9RA, United Kingdom

**Keywords:** Domestic violence, health systems readiness, violence against women

## Abstract

Domestic violence (DV) is a global prevalent health problem leading to adverse health consequences, yet health systems are often unprepared to address it. This article presents a comparative synthesis of the health system’s pre-conditions necessary to enable integration of DV in health services in Brazil, Nepal, Sri Lanka and occupied Palestinian Territories (oPT). A cross-country, comparative analysis was conducted using a health systems readiness framework. Data collection involved multiple data sources, including qualitative interviews with various stakeholders; focus-group discussions with women; structured facility observations; and a survey with providers. Our findings highlight deficiencies in policy and practice that need to be addressed for an effective DV response. Common readiness gaps include unclear and limited guidance on DV, unsupportive leadership coupled with limited training and resources. Most providers felt unprepared, lacked guidance and felt unsupported and unprotected by managers and their health system. While in Brazil most providers felt they should respond to DV cases, many in Sri Lanka preferred not to. Such organizational and service delivery challenges, in turn, also affected how health providers responded to DV cases leaving them not confident, uncertain about their knowledge and unsure about their role. Furthermore, providers’ personal beliefs and values on DV and gender norms also impacted their motivation and ability to respond, prompting some to become ‘activists’ while others were reluctant to intervene and prone to blame women. Our synthesis also pointed to a gap in women’s use of health services for DV as they had low trust in providers. Our conceptual framework demonstrates the importance of having clear policies and highlights the need to engage leadership across every level of the system to reframe challenges and strengthen routine practices. Future research should also determine the ways in which women’s understanding and needs related to DV help-seeking are addressed.

Key messages

- This is the first comparative study to assess health system readiness for domestic violence (DV) in low- and middle-income countries (LMIC).
- We present deficiencies and facilitators in policy and practice that need to be addressed for effective implementation of a health intervention to identify and respond to DV.
- This study emphasizes the importance of addressing the identified gaps in policy, leadership, provider training and women’s trust to enable effective integration of DV into health services.
- Our findings have shown the importance of nurturing the engagement of the health leadership across every level of the system and of ensuring clarity on roles and responsibilities for both health managers and clinicians.
- Future research should focus on understanding and addressing women’s specific needs and perceptions related to DV help-seeking.

## Introduction

Domestic violence (DV) against women is a global health concern and a human rights violation affecting women’s and children’s health. Globally, just over one in three (27%) ever-partnered women have been subjected to physical and/or sexual violence by an intimate partner (World Health Organization (WHO), 2021), leading to adverse health consequences for both women and their children (World Health Organization, 2013). There is an increasing focus on strengthening the role of health systems to identify and respond to survivors of DV, and exploring its readiness to do so (Hegarty *et al*., 2020; Colombini *et al*., 2022). Health systems readiness looks at how prepared systems and institutions, including providers and potential users, are to accept and implement the changes needed for the integration of new services. However, planning for integrating DV services rarely builds on the knowledge of what resources are currently available to health staff and managers, their attributes and the administration that will be managing the services. Current assessments of readiness are often limited to individual provider or service-level factors, focusing less attention to broader health systems dimensions, with few exceptions (D’Oliveira *et al*., 2020; Hegarty *et al*., 2020; Colombini *et al*., 2022).

### DV context in the study countries

DV is a major problem in all the countries in this study with a prevalence ranging from 29% in occupied Palestinian Territories (oPT) to 27% in Nepal (Ne), 24% Sri Lanka (SL) and 23% in Brazil (Br) (World Health Organization (WHO), 2021). oPT, Ne and SL have been affected by long-term political conflicts (with ongoing military occupation in oPT), which is associated with higher prevalence of violence against women (Clark *et al*., 2010; El Feki *et al*., 2017; Guruge *et al*., 2017). These four countries fall in the low- and middle-income countries group according to the World Bank country classification (The World Bank Group, 2023). In Br, the Unified Health System aims to ensure universal access to free health care through a single, publicly funded system (Coletiva *et al*., 2011). In Ne, health care services are delivered through public and the private sectors, with the government health care delivery system consisting of different levels of health facilities across various administrative tiers country (Department of Health Services, 2022). There is high level of health service coverage in oPT through government and non-government sectors (Department of Health Systems Development and Department of Information, 2018). Sri Lanka’s national health system focuses on universal health coverage, delivering free health care through state sector services (Rajapaksa *et al*., 2021).

Policy and legal contexts around DV are heterogeneous across the four countries. Br has a comprehensive regulatory framework with a law on DV since 2006 (D’Oliveira *et al*., 2020). Over the past two decades, Ne and SL have developed an overarching regulatory framework to combat violence with DV laws adopted in 2005 (SL) and 2009 (Ne) (Parliament of the Democratic Socialist Republic of Sri Lanka, 2005; Ministry of Law and Justice, 2009; Colombini *et al*., 2016; 2018). In contrast, oPT does not have any laws sanctioning DV, though there is limited guidance for multisectoral referral for DV and a National Committee for DV (Colombini *et al*., 2019).

DV governance structures also vary. Br has national and municipal guidelines for its health sector, and the municipality of São Paulo policy proposes to organize DV training, documentation, case support with a Violence Prevention Nucleus (NPV) in primary health care (PHC) facilities (Secretaria Municipal da Saúde, 2015). Ne and SL have policy frameworks that incorporate a health sector response for violence against women (VAW) (Government of Nepal, 2009; Ministry of Wome and Child Affairs, 2016). In these two countries, VAW (including DV and sexual violence) care is primarily offered at hospital levels through One Stop Centres [Mithuru Piyasa in SL and One Stop Crisis Management Centres (OCMCs) in Ne], which provide on-site medical care, basic counselling and external referral for long-term support (Government of Nepal, Ministry of Health and Population, 2015; Pathiraja *et al*., 2020; Department of Health Services, 2021). oPT has a less developed response for DV in PHC. PHC facilities were guided by National Referral Systems and Gender-Based Violence (GBV) focal points present in directorates of the Ministry of Health (MoH) as referral points, though clarity on health sector role was limited.

Countries’ structural context related to DV, legal and health system responses are summarized in Table 1.

**Table 1.** Structural context related to DV

	Br	Ne	oPT	SL
**National regulatory framework**				
Legal and policy framework (multisectoral)	Creation of Secretariat of Policies for Women (SPW, 2003), increasing specialized services to deal with DV cases (e.g. reference centres, shelters, specialized courts, Women’s Police stations) Comprehensive legal and policy framework on VAW: Maria da Penha Law (2006) National Policy to Address VAW (2011) Recent decrease in financing to VAW services, after the end of SPW (since 2016)	Legal and policy framework developed around DV (DV law since 2009) Relief funds for survivors of violence	No DV law, though some national coordination structures around DV (e.g. National Referral Systems and GBV focal points)	Fairly well-developed overarching legal and policy framework to combat VAW: Protection of Women from Domestic Violence Act 2005
**Health systems responses to DV/VAW**				
Service guidelines and protocols	Guideline for prevention and treatment of Sexual Violence against women and adolescents (1999, updated in 2005 and 2012)	Clinical protocol on GBV developed in 2015 for health facilities	No specific protocol, but clinics follow the National referral systems guideline (see above)	
Models for service provision	NPV (2015) in all care centres in São Paulo city (municipal policy), offering psychosocial support and referral to multi-agency networks	Main health response is at hospital level, through OCMCs	GBV focal points available in each Health Directorate (as external referral points) MoH developed a health response to DV in some PHC clinics in the West Bank (nurses case managers and referrals to GBV focal points)	Health sector response to VAW was developed at hospital level through: (1) ‘Mithuru Piyasa’ (One Stop Centres); (2) GBV desks, which have been implemented in a number of hospitals and clinics
Training	Training for NPV teams at PHC clinics	National training programme on health care response to GBV (primarily in hospitals, but not in ORCs)	Limited staff training and coordination of referrals. GBV focal points are in charge of training nurses case managers	No specific training on DV/VAW offered to health providers
Data information	Epidemiological Surveillance System of cases of VAW identified in health services (2003)	No information system in place	Epidemiological surveillance to report VAW cases since 2017	No information system in place
Referral and coordination	NPV teams coordinate referral to multiagency networks for DV (e.g. legal, social support, shelters)	Main referral to police and OCMCs	Referral systems in place between PHC clinics and GBV focal points, who are in charge of coordinating external referrals to police and other support services	Referral network through One Stop Centres (police, social support, shelter)

This article presents a comparative synthesis of the health system pre-conditions necessary to enable integration of DV health services in Br, Ne, SL and oPT. It offers an innovative conceptual framework to explore health system readiness for DV health services.

## Materials and methods

### Study settings

The synthesis was conducted as part of the HERA study (Healthcare response to Violence and Abuse) implemented in Br, Ne, SL and oPT. A detailed description of their national health system contexts is provided elsewhere (Paim *et al*., 2011; Keelan, 2016; Colombini *et al*., 2019; Xu *et al*., 2020; Rajapaksa *et al*., 2021; Adhikari *et al*., 2022).

Study sites varied according to their geographical location and type of facility (see Table 2 for additional information). In Br, oPT and SL, study facilities included urban public health facilities: PHC clinics in São Paulo (Br) and in oPT (Nablus, Jenin and Jerusalem), and hospitals in Colombo (SL). In Ne, study settings included mostly rural, hospital-owned, community-based basic health care facilities called Outreach centres (ORCs). SL and Ne used One Stop Centres as referral services.

**Table 2.** Brief description of HERA study settings

	Br	Ne	oPT	SL
**Number of study facilities**	8	10	4	2
**Level of care/type of facility**	PHC/Family Clinics	PHC/ORCs	PHC clinics	Tertiary care/hospitals
**Facility administration**	Private and non-profit organizations with municipality resources from the Universal Health System	Dhulikhel Hospital, a tertiary level hospital affiliated to Kathmandu University	Directorate of Health/MoH	Department of Health Services of Central Government
**Location and covered area**	Urban area of São Paulo (two different regions of the São Paulo municipality)	Seven rural areas, three urban areas in Bagmati province	Three urban areas in Nablus, Jenin and Jerusalem. One rural in Bethlehem	Urban area in the Kandy district, Central province

### Study tools and techniques

As part of HERA study, between June 2019 and August 2020, we conducted a health systems readiness assessment to explore readiness gaps within the following health systems dimensions: governance and leadership; resources and infrastructure; health service delivery; values and beliefs; health workforce and coordination and micro-level (women/clients). The conceptual framework adopted for this readiness analysis is described elsewhere (Colombini *et al*., 2022).

Table 3 presents sources of data collection and how they contributed to the readiness analysis. For the cross-country, comparative synthesis, we purposely selected a sample of multiple data sources that were co-developed to ensure cross-country comparisons were adapted for context-specificity.

**Table 3.** Data collection methods and contribution to readiness analysis

	Br	Ne	SL	oPT	Contribution to readiness analysis
**In-depth interviews with**:					
Health providers	58 (10 translated)	8 (2 translated)	18 (2 translated)	24 (1 translated)	Values and organizational culture Health provider readiness Organizational support and challenges
Health Managers	8 (2 translated)	8	6	12 (2 translated)	Values Organizational support and leadership Management at operational level
Women	20	/	20 (3 translated)	7	Women’s preparedness and trust Community support and engagement
**Focus group discussions with**:					
Women’s microfinance groups	/	4 FGD (2 translated)	/	/	Women’s preparedness and trust
Female community health volunteers	/	4 FGDs (3 translated)	/	/	Women’s preparedness and trust
**Structured facility observations**	8	10	0	4	Organizational and service delivery capabilities (facility readiness)
**Pre-PIM**	220	44	74	23	Health provider readiness

Qualitative interviews were conducted in local languages by trained researchers and took place at the study clinics (Br, Ne, oPT, SL) or a private place selected by the participants (Br, Ne). Interviews were audio recorded and subsequently transcribed and over two-thirds were translated into English to facilitate comparative analysis across countries.

Due to the Covid-19 pandemic, some interviews (with providers and other stakeholders) were conducted either online (Br = 34, Ne = 1) or via telephone (Ne = 2). Prior consent for these interviews was either audio recorded (Br) and/or via email with e-signature (Ne and Br).

Before the training intervention, a self-administered Provider Intervention Measure (PIM) questionnaire was circulated to all the participating health care providers at the study facilities (paper-based in Br, oPT, SL, tablet-based in Ne) to assess provider readiness to identify and respond to DV.

### Data analysis

Using a health systems readiness conceptual framework for DV (Colombini *et al*., 2022)—that was reviewed and adapted with the local partners—a cross-country and comparative analysis using multiple data sources was performed. Each country team analysed their data separately and key themes were subsequently discussed during regular online data analysis workshops with UK and country researchers. Summary matrices for country and across-country analyses were used to manage the data (Ritchie and Lewis, 2014). A subsample of 27 transcripts translated into English were shared with UK researchers to facilitate their participation in the data analysis discussions. In addition, each country produced a detailed summary of their main results including illustrative quotes translated into English for each of the identified themes and subthemes of the framework. Subsequently, a comparison of the combined results from the different countries was conducted by the whole research team (via virtual workshops). Reports were generated in Word tables to help explore data across countries and themes within each dimension of the framework matrix. The main similarities across the countries were then synthesized into key findings selected jointly by the research groups. PIM data from each country were descriptively summarized in a single summary table. Readiness was measured on a scale of ‘0’ to ‘2’ in SL but ‘0’ to ‘4’ in other three countries. To make it comparable across countries, we used crude conversion for the Sri Lankan readiness score by multiplying the score by 2 so that the range became ‘0’ to ‘4’. The completed questionnaire was returned to the researchers. Findings were presented descriptively as cross-tabulations and graphs.

### Ethical considerations and approvals

We followed WHO ethical and safety recommendations (World Health Organization, 2016) to ensure safety of study participants and of researchers. Participants were provided with information of local psychosocial support services if they needed further help. Ethical approvals for this study were received from the authors’ institutions.

## Results

Key overarching themes emerged from our thematic analysis and are presented according to the levels of the health systems readiness framework (Colombini *et al*., 2022), namely organizational, service, provider and micro-level. A summary of key findings is presented in Table 4.

**Table 4.** Summary of health systems readiness findings across study settings

HS Readiness findings	Br	Ne	oPT	SL
Organizational level				
Lack of clarity of DV policy guidance	+	+	+	+
Disconnection with high-level leadership	+	+	-	+
Limited DV training offered	+	+	+	+
Feeling unprotected within the health facilities	−	+	+	+
Service delivery level				
Adequate infrastructure	+	+	+	+
Limited resources (e.g. staff, time)	+	−	+	+
Unsupportive environment/feeling unsupported by clinic managers	+	−	+	+
Onsite dedicated person/service for DV	+	−	−	+
Provider readiness level				
Providers’ confusion about roles/low knowledge	+	+	+	+
Influence of personal values on DV response (e.g. victim blaming)	+	+	+	+
Fear of family retaliation	+	+	+	−
Micro level (women/clients)				
Low awareness of DV services	+	+	+	+
Low trust in health providers (fear they would breach confidentiality)	−	+	+	+
Low expectations about health providers’ ability to help with DV	+	+	−	+
Fear of victim blaming and shame	+	+	+	+

Legend: + = present/reported; − = not reported/not considered a challenge.

### Organizational-level readiness

#### Limited clarity of DV policy guidance

Despite all countries (except oPT) having national laws and policies and regulatory systems in place for DV, implementation of such policies at local level was weak and had not reached frontline providers. Findings from interviews with health providers and managers showed low awareness of the content of DV laws. In Br, where strong policy guidance on DV existed and providers were aware of the Maria da Penha DV Law, many lacked knowledge of its content and trust in its application by the justice system. Similarly, in SL, providers felt reluctant to refer women for legal action because they did not trust the legal system.

In oPT, providers expressed the need for laws to ensure DV cases are dealt with.

*We need laws … laws are very important … not only to us but to every institution … in case … help was sought the call will be answered and they will be protected*. (oPT, Nurse, Female)

Although DV guidelines were available in most facilities across the four countries—apart from Ne—findings from qualitative interviews with providers showed this lacked clarity when defining roles or guiding providers in identification and response. For instance, not all providers knew about the NPV or its role (Br), and some reported being confused on what to do for DV survivors (SL).

*We have no clear guidelines on how these DV cases should be handled. If a poison victim comes to the clinic, we know the exact steps that need to be taken to provide care. But in the case of a DV victim, we don’t know what should be done first, and what next. I believe that guidelines should be established … so that we have a sense of purpose and justification .*... (Sri Lanka, Nurse, Female)

#### Disconnection with high-level leadership

Clinic-level managers also were unclear about their roles and often felt detached from higher administrative leadership. Brazilian clinic managers reported they lacked policy guidance and felt that the demands of the Municipal Health Department were not compatible with facility priorities and capacity to respond. In Ne, health managers at Dhulikhel Hospital wrongly assumed that ORC staff knew about DV support services offered in their premises (though OCMCs).

##### Feeling unprotected within the health facilities

Feeling unprotected emerged as a recurrent theme among nurses and doctors.

*The hospital does not afford us any protection. Sometimes, only I am in this room. I am not protected from whatever happens here. Often, the women’s husbands who seek treatment from us come here and criticize us directly, telling us not to interfere in their domestic affairs. Such incidents have occurred many times. But, even under these circumstances, there is no one to protect us*. (Sri Lanka, Nurse, Female)

*We lack safety, to be honest. […] Honestly, we neither have protection at our jobs nor on personal levels*. (oPT, Doctor, Female)

This finding was also reflected in the PIM survey, where providers across all countries felt only moderately protected (Table 5).

**Table 5.** PIM survey

	Br (n1 = 210) frequency (%)	Ne (n2 = 44) frequency (%)	oPT (n3 = 23) frequency (%)	SL (n4 = 74) frequency (%)
**Feeling afraid of dealing with a DV survivor**				
Very afraid	18 (8.6)	1(2)	2 (8.7)	9 (12.2)
Moderately afraid	105 (50.0)	18 (41)	9 (39)	31 (41.9)
Not afraid	74 (35.2)	21 (48)	10 (43.4)	26 (35.1)
Not sure	11 (5.2)	4 (9)	2 (8.7)	8 (10.8)
Missing/ignored	2 (1.0)	0 (0)	0 (0)	0 (0)
**Feeling protected by organization when dealing with a DV survivor**				
Very protected	23 (11)	15 (34)	0 (0)	8 (10.8)
Moderately protected	84 (40.0)	20 (46)	5 (21.7)	25 (33.8)
Not protected	81 (38.6)	5 (11)	12 (52.1)	10 (13.5)
Not sure	21 (10.0)	4 (9)	6 (26)	31 (41.9)
Missing/ignored	1 (0.5)	0 (0)	0 (0)	0 (0)
**Possibility of talking about DV to female patients in a private & confidential space**				
Always possible	44 (21.0)	8 (18)	4 (17.4)	11 (14.9)
In most cases	105 (50.0)	27 (61)	6 (26)	29 (39.2)
Rarely	54 (25.7)	9 (21)	8 (34.7)	32 (43.2)
Never	5 (2.4)	2 (3.1)	5 (21.7)	2 (2.7)
Missing/ignored	2 (1.0)	0 (0)	0 (0)	0 (0)
**Knowledge of support services to refer female patients who are experiencing DV**				
Yes	146 (69.5)	10 (23)	15 (65.2)	52 (70.3)
No	32 (15.2)	27 (61)	7 (30.4)	18 (24.3)
Not sure	23 (11.0)	7 (16)	1 (4.3)	4 (5.4)
Missing/ignored	9(4.3)	0 (0)	0 (0)	0 (0)
**Availability of information on DV to use during consultations with women**				
Yes	94 (42.3)	5 (11)	9 (39.1)	50 (76.9)
No	41 (18.5)	34 (77)	7 (30.4)	11 (16.9)
Not sure	64 (28.8)	5 (11)	7 (30.4)	4 (6.2)
Missing/ignored	10 (5)	0 (0)	0 (0)	0 (0)

The absence of protective measures could limit providers’ ability to ask about DV (see section below). In addition, nurses in SL reported that the lack of safety protocols could also increase women’s risk of further marital conflicts.

##### Limited training and existing service delivery constraints

Findings on DV training (Table 5) showed that over a third of providers had received some form of DV training before the HERA intervention, other than in Ne (45% in Br, 39.1% in oPT and 33.8% in SL). Sex-disaggregated analysis showed a higher proportion of trained females in SL (35% of females vs 29% of males) and oPT (47% of females and 0% males). However, qualitative interviews with providers illustrated how many stated they never attended any formal training and some learned independently how to address DV—e.g. through teamwork and meetings (Br), learning on the job (Ne, SL) and using personal experience to offer advice (SL).

*I do my best to advise them on how to solve problems inside their home. Though I have not had any special training, I give solutions based on my life experience. I don’t know what I should do exactly*. (Sri Lanka, Doctor, Female)

### Service-level readiness

#### Adequate infrastructure but limited resources

The facility observations show that most facilities had sufficient infrastructure for DV response, though some challenges existed around limited private space in Br, oPT and Ne. Table 6 offers a summary of the key characteristics of study facilities.

**Table 6.** Summary of key baseline characteristics of the study facilities (taken from facility readiness checklist)

Key baseline characteristics	Br	Ne	oPT	SL
**Number of study facilities**	8	10	4	2
**Level of care/type of facility**	PHC/Family Clinics	PHC/ORC	PHC	Tertiary care/hospitals
**Facility administration**	Private and non-profit organizations with municipality resources from the Universal Health System	A private tertiary level hospital affiliated to Kathmandu University	Directorate of Health/MoH	Department of Health Services of Central Government
**Location and covered area**	Urban area of São Paulo	Seven rural areas, three urban areas in Bagmati province	4:3 urban areas in Nablus, Jenin and Jerusalem. One rural in Bethlehem	Urban, Central province
**Number of Staff (range; median)**	49–176; 82	2–9; 5	4–10; 7	634–683: 658
**Number of DV cases recorded in the last month**	81	0^a^	15	181
**Dedicated DV staff**	Yes	No	No	Yes
**DV training available**	Yes, except for two facilities	No	No (only two facilities mentioned DV training)	No
**DV identification**	Yes	Yes, in five facilities	Yes	Yes
**DV documentation and registration system**	Yes	In place in five facilities	Yes in two facilities	Yes, at Mithuru Piyasa
**Referral system (external)**	Yes, except for three facilities	Yes (OCMCs, Police)	Yes except for one facility	Yes (Police/divisional secretariat office/ social workers)
**Private consultation space**	Yes	Yes	No, except for one facility	Yes (at Mithuru Piyasa)
**Protocol/guidelines for handling DV cases**	Yes	No	Yes, except for one facility	No

a Did not have a DV documentation system in place before.

Staff rotation (Ne, Br) and chronic staff shortage at study facilities (all countries) also affected service delivery for DV as often health providers were overworked and clinics under-resourced. In SL, the Mithuru Piyasa (dedicated violence centres) were temporarily closed while its nurse or doctor were asked to perform other duties outside their DV work.

#### Feeling unsupported by health managers

Challenges undermining a supportive environment were reported across settings. Despite some facility-level managers being supportive and believing DV should be a priority (Br), findings show that most countries lacked district-level management support. Many health care providers and managers reported that DV was not seen as a high priority by their health managers, for various reasons. For instance, in Br, this was partly because of competing health problems (and the lack of performance indicators on DV), and partly because of a traditional biomedical approach giving less priority to psychosocial problems. In SL, some health care administrative officers did not prioritize DV and believed that it should be addressed by social institutions instead. DV care was not seen as part of routine practice, and providers were allowed limited time to work with survivors (no performance indicators existed for DV cases). Feeling unsupported by clinic managers was reported as a challenge in SL (concerning general staff shortages and safety) and oPT (especially concerning providers’ safety) and to a lesser extent in Br, where some health managers in one zone offered ‘passive’ support releasing providers to participate in training and DV meetings, rather than actively prioritizing a DV response and monitoring the work of the NPV.

#### Onsite dedicated person/service for DV

Having someone to go to when uncertain how to proceed with DV cases emerged as a facilitator in some settings. Only Br and SL had an on-site dedicated person (NPV staff in Br or a nurse at Mithuru Piyasa—in SL) embedded within facility structures to whom frontline providers could refer DV cases. Although offsite, oPT providers could contact MoH GBV focal points to refer women, while Nepali staff could contact OCMCs. In oPT, nurses reported seeking advice from MoH GBV focal points or from clinic managers. In Br, it was the NPV who provided guidance and support and acted as a referral point, though not all staff were aware of the NPV group. Overall, there was very limited reflective practice and monitoring—except some instances of DV PHC team meetings in Br and an informal ‘virtual group’ among providers in Ne.

Referral to external DV specialized services was available in almost all settings (see Table 6 on facility observations). However, several combined factors (Br, SL and oPT) affected external referrals to non-health services including unclear referral pathways, limited communication between health facilities and external specialized support services, low knowledge of referrals and confusion of roles across services. Providers in Br and SL also reported lack of trust in such external services as they had limited—and sometimes negative—feedback on DV cases they referred. This made them feel like there was an interruption in continuity of care (often due to geographical distance) and that the patient was ‘lost’.

*(…) where are you going to refer to? (…) What makes it difficult is not having a structured flow and not knowing officially where in the network I can refer the case. Because most of the time it seems that the health units do not talk to the other services in the network, understand?* (Brazil, Nurse, Female, NPV)

### Provider readiness level

#### Reduced readiness to identify and respond to DV

**Figure 1.**
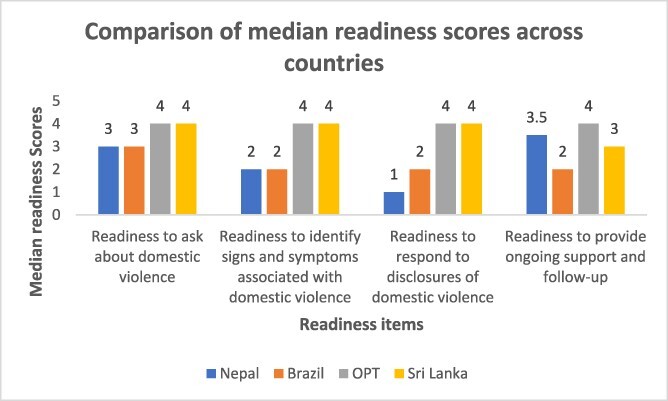
Comparison of median providers’ readiness scores across countries

PIM data on providers’ readiness (Figure 1) show how providers felt somewhat ready to ask about DV, but less prepared to identify and respond to it, apart from oPT. However, this was not fully supported by the qualitative data.

Providers’ narratives across all four countries highlighted the lack of knowledge on how to proceed, on pathways and referrals, as a major impediment to their readiness to address DV. This left them feeling less confident, unprepared, uncertain and even helpless about their roles.

#### Influence of personal values on DV response

Providers’ readiness to address DV was also influenced by personal belief and value systems on DV and their role as providers. In Br, although all providers thought that DV should be addressed by every staff member, many believed that psychologists and social workers were better equipped to do so. Similar findings emerged in SL where Health Care Providers (HCPs) reported they did not know which women to ‘interrogate’ and when they should intervene. Furthermore, that counsellors and social institutions should be solely responsible for addressing such ‘family matters’.

*I think that all professionals should be involved to a certain extent, identifying the violence, […] but I think it is very important the participation of the technical team, so the doctor, the nurse, the psychologist and the social worker*. [But the professional category that is in a better position to attend the cases are] *the psychologists and social workers […] for having a better accuracy with the psyche of the patient, for knowing how to deal better and offer more therapeutic resources for this patient who is a victim of violence*. (Brazil, Doctor, Male)

#### Personal commitment and motivation

Some showed more personal commitment and motivation informed by their belief that DV was unacceptable—and was against women’s human rights—and that dealing with DV should be part of their role (Br, Ne). In Br, some acted as ‘activists’ to ensure DV was addressed in the PHC facilities and to encourage and support other staff to act upon this issue. Even when recognized as a health issue, it was challenging for providers to include DV in their clinical practice (unless physical injuries were involved) as their expectations still tended to be curative and about ‘fixing’ the problem (through advice on divorce and reporting to the police). Other providers (many in SL, Ne, oPT) were influenced by traditional value systems that normalized gender roles and violence and saw DV as a private matter rather than a medical responsibility, underpinning their reluctance to intervene and victim blaming attitudes.

*We prioritise medical illnesses rather than the patient’s mental health. We usually treat physical injuries. We are not responsible for their family matters. Therefore, we will not make any attempt to request such information*. (Sri Lanka, Doctor, Female)

#### Fear of family retaliation

Despite having very different socio-cultural contexts, fear of family retaliation also emerged as an important theme across settings. Nearly 50% of providers were moderately or very afraid of dealing with DV cases (see Table 5). This was often interwoven with cultural normalization of DV and prioritizing the family unity, which inhibited providers from either enquiring about DV and/or documenting a case.

*Women are scared to speak to us if they are harassed by their husbands. They are scared because they must return to the same house and live together. If we ask them to do or say something, they will say that they are the ones who live in that house, not us. They don’t even want to divorce their husbands. They fear it even. So, if things are like that, how do we get involved in these cases*. (Sri Lanka, Doctor, Female)

#### Preference for female providers

Findings from SL, oPT and Ne illustrated how many health providers felt that being a female provider made it easier to inquire and more comfortable for the women to disclose DV to them. This finding was also validated by male providers who said that women preferred to disclose to female providers.

*They don’t tell us such things* [about coercive control of contraceptive use] *to us men but they might tell that to our sister* [nurse]. (Nepal, Certified Medical Assistant, Male)

*Actually, it would be better if a female physician could talk about sexual violence. They never tell anybody else. […] If we had a male doctor here, they wouldn’t say anything, even by mistake*. (Sri Lanka, Doctor, Male)

### Micro-level (women/clients) readiness

#### Women’s low engagement with DV health care responses

Our findings from interviews and Focus Group Discussions (FGDs) with women (Ne) highlighted a disconnection between women and the health care response to DV, which manifested in low awareness of DV services and distrust in DV providers. Across all settings, women did not know where to seek help following DV. Some did not think health facilities would be able to support them (Br, SL)—even though they were frequent users of the services—or thought providers were not interested in DV. Expectations from women were sometimes related to getting help for their partners’ addiction problems or their own health problems.

*I haven’t heard that such problems* [DV problems] *can be disclosed to healthcare providers … I mean, I never thought of disclosing to healthcare providers and never thought that* [I] *can get help* [from providers]. (SW13, Sri Lanka, woman)

*Such questions are rarely asked in antenatal clinics…they* [HCPs in clinics] *are mostly concerned about the children*. (SW06, Sri Lanka, woman)

Most women only knew about police stations (Ne, Br). In Ne, women only sought DV care for very severe health problems. Furthermore, the culture of silence and stigma attached to DV in Ne, SL and oPT—rooted in normative cultural values about women’s role, family unity and DV normalization—deterred women from speaking out against their husbands. Thus, most women in these settings found it hard to seek help and disclose DV to providers. While some women remained silent because of normalization of DV, others remained quiet because they considered the abuse not to be severe.

#### Women’s distrust of some health providers

In SL and Ne, women mentioned they did not always trust DV services and their providers, especially doctors because they were not familiar with them.

*It is because usually the PHM* [Public Health Midwives] *is the one who is more familiar to us. She knows about our family background. But we meet a doctor only on the day when we are getting treated or admitted to the ward*. (SW05, Sri Lanka, woman)

Many feared providers would breach their confidentiality (oPT, SL, Ne) or push them to report their husband to the police (Br). Some (oPT) also feared they would lose their children if they reported DV.

Lack of encouragement to disclose and time constraints in the health care settings (e.g. not asked about DV, lack of time to talk with providers) were also ‘service delivery’ aspects that prevented women’s disclosure in SL and Br.

#### Anticipated stigma

Shame, judgement and victim blaming by professionals were also feared by some women. In Br, shame and women’s low expectations about health providers’ ability to help them with DV prevented them from disclosing.

*I was offered to call the police or some other department, but I declined their offer because things would get out of hand, and I will be blamed and shamed for my decision*. (SW3, OPT, woman)

*I feel ashamed to disclose it* [IPV] *to them* [providers]. *I am worried about what the doctors may think of me if I tell them these things […] therefore most of the time I am trying to hide my problems*. (SW17, Sri Lanka, woman)

## Discussion

This is the first comparative study to assess health systems readiness for DV in low- and middle-income countries. The findings highlight deficiencies in policy and practice that need to be addressed for an effective response to DV. The socio-cultural realities and contexts differ significantly between Br, SL, Ne and oPT, encompassing factors such as cultural norms regarding gender roles and family structure, traditional practices related to marriage and family, religious influences shaping attitudes towards violence, socio-economic conditions affecting access to resources and support services, legal frameworks regarding DV legislation and enforcement and levels of gender equality in society, all of which intricately shape the perceptions, prevalence and responses to DV within each respective country. Despite these variations in socio-cultural realities, these policy and practice deficiencies cut across several dimensions of the health system, showing that it is not just one element that affects readiness, but intricate connections across various dimensions. For instance, challenges at organizational and service delivery levels (e.g. unclear DV guidance, lack of management support and limited training opportunities) affect providers’ responses to DV survivors, leaving them unsure of how to proceed. Similarly, barriers at provider level (e.g. low motivation, victim-blaming)—coupled with facility-level constraints (e.g. lack of time and staff)—also influence staff engagement with DV cases and subsequently DV disclosure among women.

Findings highlight some differences in readiness levels across the four settings, with Br demonstrating higher levels of readiness because of DV legislation and policies, an embedded DV structure (NPV), some organizational support and staff motivation and also staff’s widespread awareness of gender inequality rooted in a strong feminist movement. In contrast, SL, Ne and oPT still needed additional support for their staff regarding guidance, skills, motivation (regarding DV as part of their role) and organizational support (with limited leadership prioritization and engagement with DV). Despite these differences, some readiness issues cut across all settings.

Our findings corroborate the importance of having a supportive legislative framework (Klugman *et al*., 2014), but show that if its content is not institutionalized and transmitted to frontline providers and facility managers, it limits providers’ management of DV. Providers reported they lacked knowledge of policies and clear guidance on how to deal with DV cases. Some were compelled to act by their motivation, but they were not supported by the policies. The policy implementation gap—a result of the low prioritization of DV at higher level management—also affected accountability and could place women and providers at further risk for their safety. This finding is consistent with other studies (Signorelli *et al*., 2018; D’Oliveira *et al*., 2020; Colombini *et al*., 2022).

In our synthesis, the low prioritization of DV at policy level led to limited availability of resources (human, financial and time), unclear pathways of care and inadequate training for DV, which ultimately impacted on providers’ clarity on DV guidelines. Low political will among the high- and district-level health leadership also trickled down to the facility managers and provider levels. Across all our study settings, limited organizational support was a common challenge leading to providers feeling unprepared and unsupported by managers, the facility and the health system more broadly. Having someone to go to when in doubt on how to respond to DV cases emerged as an important facilitator in Br and oPT, may be because they had a defined support system in place.

Our results also show the critical need for nurturing the health leadership across every level of the system (from facility to district-level and national managers) to reframe challenges and strengthen routine practices that encourage staff engagement in DV response. If policy makers and managers do not see DV as a priority for the health system, frontline staff will be less likely to be motivated in dealing with DV (Kwamie *et al*., 2015). Furthermore, all HERA countries were characterized by top-down, hierarchical authority structures and resource constraints, both factors well-known to constrain district manager decision-making (Kwamie *et al*., 2015). The importance of policy, organizational and management support for providers is also highlighted in other studies (Colombini *et al*., 2022; Hudspeth *et al*., 2022). A systematic review on providers’ responses to DV shows that providers who collaborated within a team and worked within a supportive health organizational system were more likely to participate in identifying and responding to DV (Colombini *et al*., 2017). However, the role of management and leadership in health systems have rarely been addressed as contributors towards addressing DV (Colombini *et al*., 2019; D’Oliveira *et al*., 2020). More research is needed to understand what drives health manager’s decisions about DV and other health priorities. Further understanding is also warranted on what ‘organizational support’ and ‘supportive health leadership’ mean for providers and managers and how health facilities could become more supportive.

In the services we studied, soft skills like motivation, communication and personal beliefs on DV and gender roles affected provider’s readiness. Our findings illustrate the importance of promoting attitudes that foster gender equality and of nurturing such soft skills, as also reported in a recent review on health providers-related barriers to DV (Tarzia *et al*., 2021). Capabilities like motivation, coupled with empathy and personal commitment, emerged as key facilitators in some study settings. In Br, ‘activist’ providers were motivated because of a commitment to human rights, justice and feminist principles or personal experiences of DV. However, in many instances, health managers and providers shared the same traditional values on/towards DV (seen as a family issue and blame women for it) of the community they serve. Such views impacted the service response by preventing provider engagement in DV, which in turn limited women’s disclosure and their trust in health services, especially in oPT, Ne and SL.

Our results are in line with other studies reporting lack of training as a major challenge for DV response (García-Moreno *et al*., 2014; Taft and Colombini, 2017) impacting on providers’ lack of clarity on roles and on guidance, knowledge and self-efficacy. Historically, medical training has focused on diagnosis and treatment, a biomedical model where the power lies with the providers (Schraiber, 2012; García-Moreno *et al*., 2014; World Health Organization, 2022). This has implications for the way that providers respond to DV (more focused on treating the physical/medical injuries and thinking they need to solve the problem). It is necessary to rethink pre-service health training towards a more holistic approach that considers gender inequality and other social determinants of (women’s) health and is patient-centred. Care for DV should prioritize autonomy, negotiation, empathy and listening that is responsive to women’s needs (World Health Organization, 2022).

Multisectoral coordination plays a crucial role in responding to DV (García-Moreno *et al*., 2014). Nonetheless, our research has underscored the difficulties encountered in collaborating with external agencies. Health care professionals in Br and SL, where external referral systems are organized through NPV and One Stop Centres, expressed reservations about external social support services due to inadequate communication with them. They often received limited, and sometimes unfavourable, feedback on the DV cases they referred. Additional research is necessary to explore ways to enhance the connections between the health care and other systems in supporting DV responses.

Lastly, our analysis shows low engagement of women/community with health services for DV, though reasons vary. In oPT, it was due to a lack of trust in providers being able to protect confidentiality (where fear of family retaliation is high), while in SL, women also feared providers’ judgement. In contrast, some women in Br and SL thought that providers would not be interested and that PHC services would not deal with DV as it was not a conventional health issue. Such misconceptions may prevent women from seeking help in DV situations (Colombini *et al*., 2022; Silva *et al*., 2022). Low trust in providers was also influenced by the stigma around DV and its normalization within health systems, which is part of the structural barriers for addressing DV (Hudspeth *et al*., 2022). This was particularly evident in oPT, Ne and SL, where prevailing social norms favour family unity and normalize DV leading to social stigma for women who report DV.

Despite global calls for women-centred and people-centred approaches in the health systems and violence literature (World Health Organization (WHO), 2015; World Health Organization, 2022), most DV health interventions have failed to closely connect with women and communities (Mannell *et al*., 2016; Colombini *et al*., 2022). PHC health providers’ proximity to the community they serve could be a facilitator for reaching women—as seen in Br (Signorelli *et al*., 2018). To strengthen the linkages between community and health-based services, future DV research should co-develop practices for substantively engaging with women and communities and improve their awareness and trust in services and providers.

### Strengths and limitations

A strength of this synthesis study is the use of a health systems readiness approach, which led to identification of facilitators and bottlenecks and their interconnections at different system levels. Having diverse data sources also helped with data triangulation and credibility. Limitations include variation in the scale measuring provider readiness across the countries. Except Ne, our study did not include discussions with community members about DV norms and beliefs. However, we used interviews with women and providers as a proxy to explore such beliefs.

## Conclusion

Our study has identified anticipated readiness gaps and facilitators specific to the capacity to address DV that are common across the four study settings, despite their diverse social and cultural realities. Our method revealed common patterns and insights that can stimulate reflection on systems readiness in other settings. Future research should also determine the ways in which women’s understanding and needs related to DV help-seeking are addressed.

## Data availability

The data (without identification) is available from the corresponding author upon reasonable request (and approval from country partners).

## Reflexivity statement

This article presents research conducted by a diverse group of researchers in terms of gender identity, seniority and citizenship. This article includes 17 authors: 13 who identify as women and 4 who identify as men. A total of 8 of the authors are early career researchers. In all, 4 of the authors are affiliated with high-income country institutions, while the remainder are affiliated with low- and middle-income countries institutions. The majority of the research and writing team includes authors with diverse cultural heritages and originating predominantly from low- and middle-income countries—and from the four study countries. The research team has multidisciplinary expertise in health systems, policy analysis, clinical medicine, public health and social science. All authors have extensive experience with qualitative research and health systems and service research and dynamics in the Global South.
